# Integration of Traditional and Metabolomics Biomarkers Identifies Prognostic Metabolites for Predicting Responsiveness to Nutritional Intervention against Oxidative Stress and Inflammation

**DOI:** 10.3390/nu9030233

**Published:** 2017-03-04

**Authors:** You Jin Kim, Iksoo Huh, Ji Yeon Kim, Saejong Park, Sung Ha Ryu, Kyu-Bong Kim, Suhkmann Kim, Taesung Park, Oran Kwon

**Affiliations:** 1 Department of Nutritional Science and Food Management, Ewha Womans University, Seoul 03760, Korea; eugene841226@gmail.com; 2 Department of Statistics, Seoul National University, Seoul 08826, Korea; huhixoo@gmail.com; 3 Department of Food Science and Technology, Seoul National University of Science and Technology, Seoul 01811, Korea; jiyeonk@seoultech.ac.kr; 4 Department of Sport Science, Korea Institute of Sport Science, Seoul 01794, Korea; saejpark@sports.re.kr; 5 College of Pharmacy, Dankook University, Chungnam 31116, Korea; shryu@glpt.co.kr (S.H.R.); kyubong@dankook.ac.kr (K.-B.K.); 6 Department of Chemistry and Chemistry Institute for Functional Materials, Pusan National University, Busan 46241, Korea; suhkmann@pusan.ac.kr

**Keywords:** oxidative stress, inflammation, prognostic marker, metabolomics, sedentary overweight/obese adults

## Abstract

Various statistical approaches can be applied to integrate traditional and omics biomarkers, allowing the discovery of prognostic markers to classify subjects into poor and good prognosis groups in terms of responses to nutritional interventions. Here, we performed a prototype study to identify metabolites that predict responses to an intervention against oxidative stress and inflammation, using a data set from a randomized controlled trial evaluating Korean black raspberry (KBR) in sedentary overweight/obese subjects. First, a linear mixed-effects model analysis with multiple testing correction showed that four-week consumption of KBR significantly changed oxidized glutathione (GSSG, *q* = 0.027) level, the ratio of reduced glutathione (GSH) to GSSG (*q* = 0.039) in erythrocytes, malondialdehyde (MDA, *q* = 0.006) and interleukin-6 (*q* = 0.006) levels in plasma, and seventeen NMR metabolites in urine compared with those in the placebo group. A subsequent generalized linear mixed model analysis showed linear correlations between baseline urinary glycine and N-phenylacetylglycine (PAG) and changes in the GSH:GSSG ratio (*p* = 0.008 and 0.004) as well as between baseline urinary adenine and changes in MDA (*p* = 0.018). Then, receiver operating characteristic analysis revealed that a two-metabolite set (glycine and PAG) had the strongest prognostic relevance for future interventions against oxidative stress (the area under the curve (AUC) = 0.778). Leave-one-out cross-validation confirmed the accuracy of prediction (AUC = 0.683). The current findings suggest that a higher level of this two-metabolite set at baseline is useful for predicting responders to dietary interventions in subjects with oxidative stress and inflammation, contributing to the emergence of personalized nutrition.

## 1. Introduction

ROS overproduction and subsequent low-grade inflammation are believed to be reasons for the acceleration of age-related chronic diseases [1]. Epidemiological evidence has indicated that foods and their constituents have been associated with reducing oxidative stress and inflammation, thus implicating them in preventing the onset of chronic disease [2,3]. However, many randomized human intervention studies to assess the benefits of foods or food constituents have often led to negative results. It is partly because differences between study subjects may be much larger than differences directly related to nutritional intervention [4]. To address this issue, the concept of precision nutrition or personalized nutrition has been introduced, where the understanding of individual’s response to an intervention is required to be achieved [5]. Fortunately, advances in omics technologies and statistical analysis have now begun to make it possible to obtain holistic and systemic information from even a single nutritional intervention study, thus making precision nutrition a realistic goal [6,7]. 

Particularly, metabolomics are known as powerful and sensitive tools that can reveal crucial information that is closely related to an individual’s current health status and responses to nutritional interventions [8]. The promising field of metabolomics involves the estimation of exposure to specific foods, such as methylglutarylcarnitine for cocoa [9], proline betaine for citrus [10], resveratrol for wine [11], 2-furoylglycine for coffee [12], alkylresorcinols for whole grains [13], and furan fatty acids (3-carboxy-4-methyl-5-propyl-2-furanpropionic acid) for fish [14], to name a few examples. Alternatively, metabolomics approach may be useful for the development of precision nutrition. To accomplish precision nutrition, the identification of desired health outcomes and valid biomarkers to measure how response changes are critically important. Moreover, biomarker should be sufficiently accurate and have a relationship in the predicted direction [15]. It has been portrayed that an appropriate prognostic markers will be useful to predict future response and enable target interventions to those who need or respond to them [5]. At present, however, only a few studies have focused on the use of metabolomics in the search for prognostic markers [16,17]. 

Black raspberry is one of the most economically important crops and has become a popular food because it is a rich source of vitamins and polyphenolic compounds with high antioxidant capacities [18]. Black raspberry is a common name for three *Rubus* species: *Rubus coreanus*, native to Eastern Asia; *Rubus occidentalis*, native to Midwestern and Eastern North America; and *Rubus leucodermis*, native to the Pacific Northwest [19]. In Korea, *Rubus coreanus* (Korean black raspberry (KBR)) and *Rubus occidentalis* (Northern American black raspberry (NAB)) have been widely used with confusion. Two recent studies compared bioactive component in KBR and NAB using fingerprinting techniques and revealed that each varied in proportions and total concentration of bioactive components [20,21]. However, thus far a comparison of biological effect between KBR and NAB remains poorly understood in a clinical setting, providing a rationale to initiate a human intervention study.

In this work, we first conducted a preliminary study to compare the antioxidative and anti-inflammatory properties of KBR with those of NAB in a randomized controlled trial with sedentary overweight/obese adults challenged with treadmill exercise at 60% VO_2_ maximum for 30 min. This approach is based on the previous findings, which indicated that acute moderate exercise is known to induce transient inflammation and oxidative stress [22]. Then, we performed a prototype study to explore the practical use of metabolites as a method for classifying subjects into poor and good prognosis groups in response to a nutritional intervention. To this end, we obtained ^1^H-NMR metabolomics data derived from the KBR group and applied various statistical analyses to integrate traditional and metabolomics biomarkers. 

## 2. Materials and Methods 

### 2.1. Test Materials 

Freeze-dried powder of KBR and NAB and a color/flavor-matched placebo containing lactose were provided by the Korean Rural Development Administration (Suwon, Gyunggi-do, Korea). The full chemical signatures and in vitro antioxidant capacities of KBR and NAB have been reported in previous publications [23,24]. The daily dose (30 g/day; equivalent to 100 g/day of fresh fruits) was determined based on previous studies from other researchers [25,26]. The daily dose of KBR represented 0.9 g of total phenol, including 17.5 mg of myricetin, 9.6 mg of genistin, 7.2 mg of quercetin, 1.2 mg of daidzein, and 1.2 mg of eriodictyol, as well as 126 kcal (65.5% as carbohydrate, 10.1% as protein, and 5.4% as fat). The daily dose of NAB represented 1.3 g of total phenol, including 25.2 mg of myricetin, 16.6 mg of genistin, 7.4 mg of kaempferol, 3.9 mg of quercetin, 1.8 mg of eriodictyol, and 0.6 mg of daidzein, as well as 111 kcal (67.8% as carbohydrate, 7.5% as protein, and 7.6% as fat) [21]. 

### 2.2. Participants

One hundred and two subjects (30–60 years) with a body mass index (BMI) between 23 and 30 kg/m^2^ and a sedentary lifestyle were recruited from the general public by poster advertisements. According to the recommendation of World Health Organization for Asian populations, 82% of subjects were classified as overweight (23–27.5 kg/m^2^) and 18% as obese (>27.5 kg/m^2^) [27]; and according to the recommendation of the Institute of Medicine, all subjects were classified as sedentary (2.5 h of exercise/week) [28]. The exclusion criteria included the current use of dietary supplements; inflammatory disease, liver disease, renal disease, cardiovascular disease, hypertension, stroke, diabetes mellitus, or any other disease affecting the results of the study; difficulty engaging in treadmill exercises; cigarette smoking; known hypersensitivity to the study product; and pregnancy or lactation. After providing written informed consent, participants underwent anthropometric measurements, a complete blood count analysis, and an exercise treadmill test to evaluate their eligibility status. The maximal oxygen consumption (VO_2max_) and maximum heart rate were determined during an incremental exercise program (2% grade increase every 2 min at a constant pace, 5–8 km/h) using a treadmill (T150; COSMED, Albano Laziale, Rome, Italy) and a respiratory gas analyzer (Quark CPET; COSMED, Albano Laziale, Rome, Italy). 

### 2.3. Experimental Design 

Seventy-two eligible subjects were enrolled at Ewha Womans University, which had three arms (placebo, KBR, and NAB). During a two-week lead-in period, participants were recommended to maintain their usual dietary and exercise habits and to avoid high-flavonoid foods and beverages including berries, fruits, vegetables, juices, microalgae, and teas for minimizing between-subject variability of bioactive components at baseline. Upon completion of a two-week lead-in period, subjects were randomly assigned to each group for 14 days using computer-generated random numbers at a ratio of 1:1:1 via stratified block randomization. Investigators and participants were blind to group allocation. During the treatment period, subjects were required to consume one sachet of corresponding test materials before each meal. Subject compliance was assessed by counting returned sachets and questioning the subjects. Changes in dietary habits and physical activity were monitored using a three-day (two weekdays and one weekend day) dietary record and were analyzed using a computerized nutritional analysis program (Can-Pro 3.0, The Korean Nutrition Society, Seoul, Korea). 

At baseline and at the end of the trial, a treadmill exercise challenge was administered for 30 min at 60% VO_2max_ to perturb the subject’s homeostasis and then to quantify the responsiveness to test materials. Venous blood samples were collected in K2-EDTA-coated tubes (Becton Dickinson, Franklin Lakes, NJ, USA) before and immediately after the completion of exercise, followed by the separation of erythrocytes from plasma by centrifugation at 1500× *g* for 10 min. Spot urine samples were collected in polypropylene containers before and immediately after the completion of exercise. The samples were stored at −70 °C until analysis. 

The study protocol was approved by the Institutional Review Boards of Ewha Womans University (Seoul, Korea) and was registered with the WHO International Clinical Trials Registry Platform under the following identification number: KCT0000644. 

### 2.4. Measurements of Traditional Biomarkers 

Plasma malondialdehyde (MDA; intra-Coefficients of Variance (CV): 13.1%; inter-CV: 5.2%) levels were determined by HPLC fluorescence (emission = 515 nm, excitation = 553 nm; SHISEIDO, Tokyo, Japan) with a Capcell Pak C18 column (UG120 type, 5 μm × 4.6 mm × 150 mm, Shiseido). Plasma oxidized LDL (ox-LDL; intra-CV: 7.9%; inter-CV: 9.6%), interleukin-6 (IL-6; intra-CV: 7.4%; inter-CV: 7.8%), and tumor necrosis factor-α (TNF-α; intra-CV: 4.9%; inter-CV: 7.6%) were measured with ELISA kits (Mercodia, Uppsala, Sweden for ox-LDL; R&D Systems, Minneapolis, MN, USA for IL-6 and TNF-α). Reduced (GSH; intra-CV: 4.0%; inter-CV: 5.9%) and oxidized glutathione (GSSG; intra-CV: 9.9%; inter-CV: 4.9%) levels in erythrocytes were measured as described by Rahman et al. [29]. Erythrocyte antioxidant enzyme activities (glutathione peroxidase, GPx (intra-CV: 5.7%; inter-CV: 7.2%); superoxide dismutase, SOD (intra-CV: 3.2%; inter-CV: 3.7%); and catalase, CAT (intra-CV: 3.8%; inter-CV: 9.9%)) and total hemoglobin (Hb) were measured spectrophotometrically using commercially available kits (Cayman, Ann Arbor, MI, USA). All measurements were performed in duplicate. 

### 2.5. ^1^H NMR Spectroscopy and Pre-Processing of NMR Spectra 

After the plasma and urine samples were thawed and centrifuged, aliquot of each sample was transferred to a microcentrifuge tube containing phosphate buffer and deuterium oxide with 0.05% 3-(trimethylsilyl)-propionic-(2,2,3,3-d4) acid sodium salt as an internal standard for plasma and 2,2-dimethyl-2-silapentane-5-sulfonate for urine. Each sample was vortexed for 60 s and centrifuged for 10 min at 7000 rpm, and then an aliquot was used for analysis. 

^1^H-NMR spectroscopy was conducted on a Varian 600 MHz spectrometer (Varian, Palo Alto, CA, USA) at Pusan National University (Pusan, Korea). One-dimensional NMR spectra were acquired with the following parameters: spectral width 24,038.5 Hz, 3 s acquisition time, and 128 nt. Additional conditions of a relaxation delay time of 1 s and a saturation power of 4 were set to suppress massive water peaks. NMR spectra of each sample were acquired once since NMR is a highly reproducible technique [30]. NMR spectra were reduced to data using the Chenomx NMR Suite program 7.6 (Chenomx, Edmonton, AB, Canada). The spectral ^1^H NMR region of δ 0.0–10.0 was segmented into regions with a width of 0.04 ppm, providing 250 integrated chemical shift regions in each NMR spectrum. The spectral regions corresponding to water (δ 4.5–5.0) were removed before normalization and spectra alignment. Metabolite concentrations were annotated and quantified manually in the NMR spectra using the Chenomx NMR Suite Professional software package 7.6 (Chenomx). For urine samples, metabolite concentrations were adjusted to the creatinine concentration because spot urine measurements, rather than 24 h urine samples, were used in this study [31]. 

### 2.6. Statistical Analysis 

The sample size was estimated at 24 subjects per group to provide a power of 80% to detect a difference in GPx activity based on a previous KBR study [26] with a two-sided α-level of 0.05, allowing for an attrition rate of 20%.

Skewed data were logarithmically or square root transformed, but the results are expressed as the arithmetic means ± standard errors of the mean (SEMs) for ease of understanding. SAS 9.3 (SAS Institute, Cary, NC, USA), and the glmer function of the lme4 package [32], the heatmap.2 function of the gplots package [33], and the auc function of the pROC package [34] in R were used for the analysis. 

Differences in means for the traditional and metabolomics biomarkers were analyzed using a linear mixed-effects (LME) model, taking into account a random effect (participant), a random error (within-participant), fixed effects (group, week, and the interaction between group and week), and a covariate (exercise). Corrected *p*-values (*q*-values) were calculated using Storey’s false discovery rate (FDR) approach (95% confidence intervals) [35,36] to correct for multiple testing. An expected pathway of differential metabolites was drawn according to the Kyoto Encyclopedia of Genes and Genomes website (http://www.genome.jp/kegg/) and the MetaCyc Encyclopedia of Metabolic Pathways (http://www.metacyc.org/) and referring to Zgoda-Pols JR et al. [37]. Then, to explore prognostic metabolites for classifying the subjects into poor and good prognosis groups in terms of oxidative and inflammatory stress, a generalized linear mixed (GLM) model was applied to the data set with a logit link function and backward elimination optimization. In this model, ^1^H NMR baseline metabolites were dichotomized (coded as 1 or 0) using median value and changes in traditional biomarkers were dichotomized to reflect positive or negative responses. Correlations between the variables were visualized using a heat map scaled by the t-value of GLM. The accuracy of predictions was tested by an area under the receiver operating characteristics (ROC) analysis. ROC curves were obtained by plotting the true-positive rates (sensitivity) against the false-positive rates (1-specificity). Areas under the curve (AUCs) with 95% CIs were calculated for sensitivity and specificity values. *P*-values were calculated for the comparison of the area under the ROC curve of each model with the reference line of 0.5. Finally, the validation of the most likely prognostic marker was performed with a leave-one-out cross-validation (LOOCV) technique. 

## 3. Results

### 3.1. Preliminary Study to Compare Oxidative Stress and Inflammation in the KBR and NAB Groups Using Traditional Biomarkers

A total of 72 subjects were enrolled and 67 subjects were evaluable for response in a preliminary study (Supplemental Figure S1). All the participants were documented to fit the protocol and the groups were well matched for age and sex (Supplemental Table S1). From the three-day dietary records completed during the intervention, no significant group effect was detected across the baseline and four-week intervention among the dietary intake variables in terms of calories or micronutrients (Supplemental Table S2). The overall compliance was estimated at 96%. No serious or severe adverse events were observed. 

The MDA, oxidized LDL, TNF-α, and IL-6 were measured in plasma. However, endogenous antioxidants and enzymes including GSH, GSSG, GPx, SOD, and CAT were measured in erythrocytes, because they are abundant in erythrocytes, which are constantly subjected to oxidative stress. The data demonstrated that the overall effect was similar for both KBR and NAB, but KBR showed a more significant effect than NAB in terms of GSSG (*q* = 0.036), GSH:GSSG (*q* = 0.050), and MDA (*q* = 0.008) levels (Supplemental Table S3). 

### 3.2. Selection of Traditional Biomarkers and Metabolites for Integration 

The ^1^H-NMR metabolomics data were obtained from the KBR group: 63 metabolites were identified in the urine samples (Supplemental Table S4) and the 31 metabolites were identified in the plasma samples (Supplemental Table S5). A LME model was used to assess differences in the KBR and the control group. As a result, four traditional biomarkers and sixteen urinary metabolites with FDR *q*-values less than 0.05 were selected to be included in further analysis (Table 1). Four traditional biomarkers were GSSG (*q* = 0.027) and GSH:GSSG ratio (*q* = 0.039) in erythrocytes and MDA (*q* = 0.006) and IL-6 (*q* = 0.006) in plasma; and sixteen metabolites were amino acids (alanine, asparagine, glutamine, glycine, histidine, lysine, serine, and carnitine), organic acids (citrate and formate), purine nucleotide (adenine), and other metabolites (N6-acetyllysine, betaine, 3-indoxylsulfate, *N*-phenylacetylglycine (PAG), and phenylacetate). 

**Figure 1:**
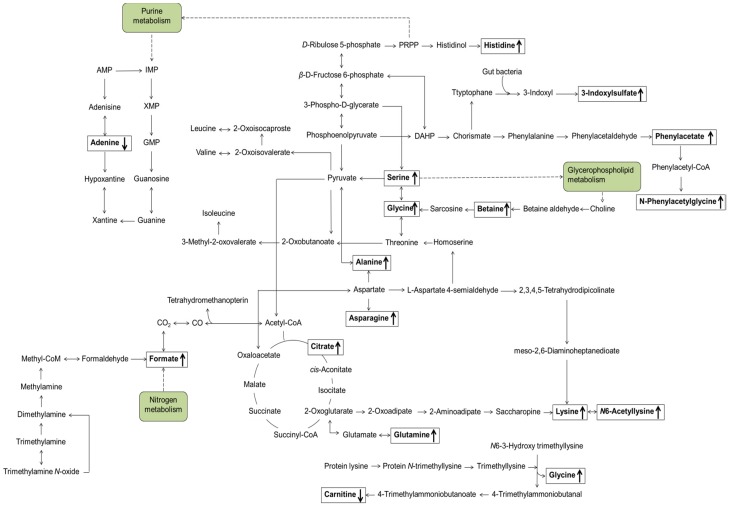
Proposed metabolic pathways related to endogenous urinary metabolites that were significantly changed in response to KBR administration over four weeks compared with those in the placebo group. Arrows indicate the directions of alterations. KBR, Korean black raspberry.

Pathway analysis using KEGG database identified the tricarboxylic acid (TCA) cycle/oxidative phosphorylation (citrate, formate, and glutamine), glycerophospholipid metabolism (serine, betaine, glycine, and choline), purine metabolism (adenine, glutamine, and glycine), and amino acid metabolism (alanine, asparagine, aspartate, glutamine, glycine, histidine, isoleucine, leucine, lysine, and serine) (Figure 1). 

### 3.3. Identification of Candidate Prognostic Metabolites 

**Figure 2:**
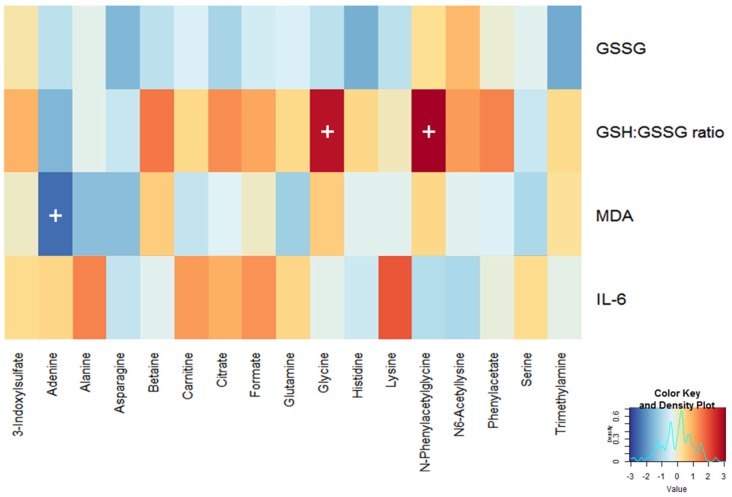
Correlation heat map generated by a generalized linear mixed model analysis of four traditional biomarkers with seventeen urinary metabolomic signatures. Red and blue colors indicate negative and positive t-values, respectively. A cross indicates a *p*-value < 0.05.

The selected biomarkers were integrated to identify associations between alterations in four traditional biomarkers and those in seventeen urinary metabolites at baseline using a GLM analysis. The key statistical measures (Supplemental Table S6) and the resulting heat map (Figure 2) revealed that urinary glycine and PAG levels were positively associated with an increase in the erythrocyte GSH:GSSG ratio (*p* = 0.008 and 0.004, respectively). In contrast, the urinary adenine level was negatively associated with a decrease in the plasma MDA level (*p* = 0.018).

### 3.4. Validation of Prognostic Metabolites 

**Figure 3:**
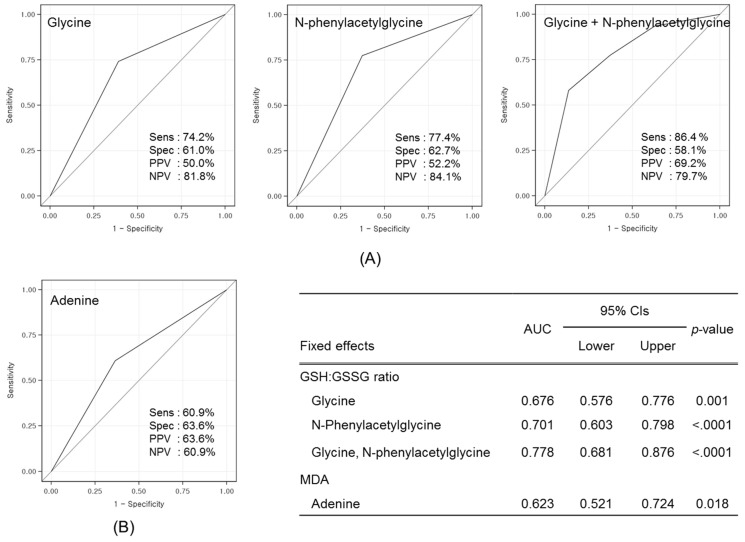
ROC curves of three single metabolite and a two-metabolite set for predicting changes in traditional biomarkers: (**A**) erythrocyte GSH:GSSG ratio; and (**B**) plasma MDA level. The gray diagonal line represents the reference line of 0.5. Sensitivity, specificity, PPV, and NPV are shown in each box, and AUC, CI, and *p*-value are presented in the inset. ROC, receiver operating characteristic; GSH, glutathione; GSSG, oxidized glutathione; MDA, malondialdehyde; PPV, positive predictive value; NPV, negative predictive value; AUC, area under the curve; CI, confidence intervals.

**Figure 4:**
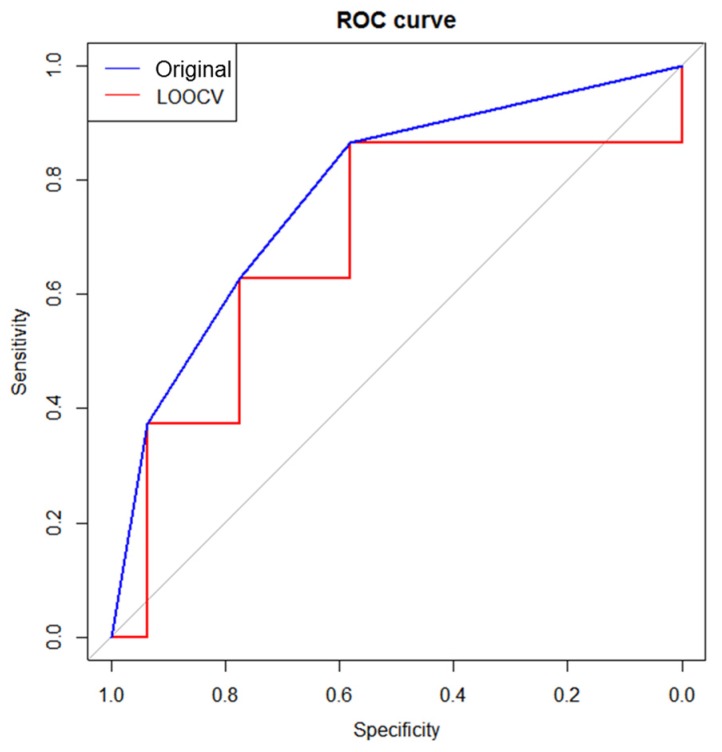
ROC curve of a two-metabolite set (glycine + N-phenylacetylglycine) by LOOCV. The blue and red ROC curves were generated using the original data set and the LOOCV data set. The gray diagonal line represents the reference line of 0.5. LOOCV, leave-one-out cross-validation.

An ROC analysis was performed on the three single candidate metabolites (glycine, PAG, and adenine) and a two-metabolite set (glycine + PAG) to test the prognostic performance (Figure 3). A two-metabolite set demonstrated the highest prognostic value, with a sensitivity of 86.4% and a specificity of 58.1% (AUC = 0.778, *p* < 0.0001). Therefore, the predictive ability of this two-metabolite set was further validated using an LOOCV analysis, demonstrating an AUC of 0.683 with a sensitivity of 86.4% and a specificity of 58.1% (Figure 4). 

## 4. Discussion

This study was composed of two parts. We first performed a preliminary study to compare anti-oxidative/anti-inflammatory properties of KBR with those of NAB in a clinical setting. Participants in the study were sedentary overweight/obese subjects presented with an exercise challenge because existing data indicate that such individuals are vulnerable to oxidative stress and have low-level chronic inflammation [38]. Using metabolic profiling, Park et al. [21] showed that myricetin, genistin, quercetin, daidzein, eridictyol are the major components both in KBR and NAB, but the proportions and contents of individual components were different each other. Based on fingerprinting of anthocyanins, Lee et al. [20] demonstrated that cyanidin-3-glucoside, cyanidin-3-rutinoside, and pelargonidin-3-glucoside were three major components in KBR, while cyanidin-3-sambubioside and cyanidin-3-xylosylrutinoside were major components in NAB. However, a bioassay for comparing the anti-oxidant capacities of KBR and NAB in vitro revealed that the level of general antioxidant activities of KBR was indistinguishable from that of NAB [24]. In this clinical trial, we demonstrated a consistent result that KBR and NAB had an almost equivalent efficacy on oxidative stress and inflammation, although KBR exhibited more or less superior activities to NAB in terms of GSH:GSSG ratio, MDA, and IL-6 levels. Taken together, we could conclude that the distinct profiles of bioactive components can be used as marker compounds to confirm the identity between KBR and NAB, but may not be good enough to be used as exclusive active components for protecting oxidative stress and inflammation.

Second part was the main study, in which we performed a pioneering study to explore whether baseline levels of metabolites in biofluids would predict a change in traditional biomarkers related to oxidative stress and inflammation in response to a nutritional intervention on an individual level. Two biofluids, plasma and urine, were used to collect relevant information on endogenous metabolites. Urine samples are known to be advantageous to study due to the ease of sample collection, large sample volumes, high concentrations, and few interfering proteins [39,40], implicating that the urinary metabolites could be more useful for identifying individuals who are at risk for progression of disease [41]. In the present study, we demonstrated that urinary metabolites were more responsive to KBR administration than plasma metabolites. 

Endogenous metabolites are quite different from food metabolites in terms of characteristics and applications [42]. Endogenous metabolites are defined as low-molecular-weight chemicals derived from the host, and provide a rich source of information regarding physiological responses to foods or their constituents. Thus, endogenous metabolites are useful for either discriminating responders from non-responders who are likely to benefit from a nutritional intervention [16,17] or predicting effectiveness for maintaining or improving health at the individual level [43]. In contrast, food metabolites are defined as chemicals derived from the digestion, absorption, and biotransformation of foods, and thus can be used for the accurate monitoring of food exposure [44]. Among the analytical techniques that can be employed for the quantitative detection of multiple metabolites in biofluids, NMR spectroscopy and mass spectrometry are the most common. ^1^H NMR spectroscopic techniques have inherently low sensitivity; thus, they cannot detect components at low concentrations below the range of micromoles per liter [42]. The major bioactive components found KBR are rapidly eliminated or transformed, thus it is not possible to detect them in biofluids collected after overnight fasting, thus metabolites cannot be used to define compliance [45]. However, NMR spectroscopy allows the detection of a wide range of endogenous metabolites. In this study, a total of 63 metabolites in urine samples and 31 metabolites in plasma samples were detected. Of these, 26 metabolites (3-hydroxybutyrate, acetate, acetone, alanine, arginine, betaine, choline, citrate, creatine, formate, glucose, glutamine, glycerol, glycine, histidine, isoleucine, lactate, leucine, lysine, methanol, phenylalanine, pyruvate, serine, succinate, tyrosine, and valine) were detected in both biofluids. 

To identify candidate prognostic metabolites and enhance the prognostic power, various statistical approaches were used. First, signature biomarkers were identified, which were then integrated to obtain associations between baseline metabolites and changes of traditional biomarkers. Similar approaches were employed to identify plasma metabolite signatures for predicting glucose tolerance changes in sedentary women after high-intensity interval training [17] and to develop predictive models for prostate carcinoma recurrence [46]. However, to our knowledge, no previous study has investigated the use of prognostic markers for screening potential responders to maximize the benefits of nutritional intervention against oxidative stress. In addition, we tested whether changing the baseline metabolites could increase the diagnostic validity by ROC analysis. The ROC curve for a two-metabolite set (glycine and PAG) outperformed single markers (glycine, PAG, or adenine), highlighting that this set is a promising biomarker that possesses the greatest discriminatory power to predict individual responses to KBR consumption in sedentary overweight/obese adults. Several other studies have supported an association between urinary glycine or PAG levels and status of oxidative stress [47,48,49,50,51,52] or inflammatory stress [52]. Finally, the predictive accuracy of this two-metabolite set was further validated with the LOOCV procedure, because the ROC curve may lead to an overestimation of the AUC if it is constructed using all samples [53]. The LOOCV procedure has an advantage of reducing the likelihood of developing an overly optimistic predictive model given the relatively small sample in our study [54]. As a result, we concluded that the predictive accuracy was fair, indicating that a higher level of glycine and PAG set may serve as a prognostic marker for planning strategic personalized interventions on oxidative stress and inflammation. 

Metabolic pathway analysis revealed that urinary levels of glycine, PAG, and adenine are involved in purine [55,56] and phenylalanine metabolism [57], conceivably resulting in changes in the GSH redox state and suppression of oxidative stress. Glycine is a precursor to GSH, thus indirectly contributing to the role of a cytoprotective agent by ROS scavenging mechanisms [48,58]. In the case where glycine availability is reduced, for example by protein malnutrition, sepsis, and diabetes, reduced glycine availability may become a limiting factor for GSH synthesis [59]. A couple of studies supported the notion that a high level of urinary glycine is associated with accelerating GSH restoration against the oxidative stress [48,58,60]. PAG is a glycine conjugate, which is expected to respond in the same direction with glycine [50]. In contrast, however, when adenine is present in excess, xanthine oxidase is activated, inducing oxidative stress by hydroxyl free radicals and hydrogen peroxide [61]. It was also reported that excessive adenine found in a chronic renal failure animal model was related to oxidative damages and inflammation [55,62]. Collectively, these data may explain our finding that background levels of three urinary metabolites were associated with internal capacity to overcome oxidative stress and thus useful to differentiate responders from non-responders to a nutritional intervention as prognostic markers.

It is important to note the limitations of this study. Firstly, for validation of the predictive ability of a proposed metabolite, we used the LOOCV procedure. However, it could not be a substitute for external validation on an independent sample set. Therefore, the next step will be to validate this result in a large cohort or other clinical studies. Secondly, in the present study, we only suggested that the subjects who have a statistically higher mean value of background urinary glycine and PAG levels may have a good prognosis following a nutritional intervention against oxidative stress. Further evidence seems to be necessary for establishing the cut-off point for the examination, rather than the performance of the test as a whole. Lastly, translational works are needed to gain future insights into the use of a two-metabolite set as a simple laboratory diagnostic kit for identifying responders to create personalized nutritional interventions that maximize the salutary benefits on an individual level. However, even with these limitations, the model we have proposed may provide fundamental understanding of the process and insight in developing prognostic metabolites for identification of potential responders who can magnify the salutary benefits of nutritional intervention against oxidative stress on an individual level. Afterwards, when aiming at an antioxidant intervention, subjects would be selected using the proposed prognostic markers to implement the individually tailored nutritional intervention. 

## 5. Conclusions

The metabolites may not only be limited to assessing overall dietary exposure, but also may be useful when selecting individuals who are most likely to benefit from nutritional interventions. The results obtained in this study provided insight into the opportunities involved in identifying prognostic metabolic markers, which may be useful for classifying responders and non-responders to nutritional interventions in subjects with oxidative stress and inflammation. Together with further studies, these results may be contributing to the emergence of personalized nutrition. 

## Figures and Tables

**Table 1:** Significantly altered traditional metabolomics biomarkers in response to KBR consumption in sedentary overweight/obese adults ^1^.

Variables	Placebo		KBR		β ^2^	*q*-Value ^3^
	Baseline	Delta Change	Baseline	Delta Change		
Traditional biomarkers						
GSSG (µM/g Hb)	12.9 ± 0.6 ^4^	1.8 ± 0.3	12.9 ± 0.7	0.3 ± 0.4	−1.117	0.027
GSH:GSSG ratio	3.0 ± 0.2	−0.3 ± 0.1	2.8 ± 0.2	0.2 ± 0.2	0.045	0.039
MDA (nM)	14.5 ± 1.5	0.0 ± 0.4	16.4 ± 1.6	−2.5 ± 0.6	−0.058	0.006
IL-6 (pg/mL)	196.1 ± 22.8	51.4 ± 31.8	182.7 ± 24.4	−52.1 ± 17.9	−0.199	0.006
Urinary metabolites (μM)						
3-Indoxylsulfate	2.31 ± 0.16	0.11 ± 0.21	2.16 ± 0.18	0.81 ± 0.2	0.399	0.009
Adenine	1.26 ± 0.11	0.09 ± 0.18	1.64 ± 0.21	−0.6 ± 0.2	−0.286	0.041
Alanine	2.79 ± 0.18	−0.14 ± 0.17	2.49 ± 0.16	0.35 ± 0.13	0.194	0.021
Asparagine	1.62 ± 0.09	0.16 ± 0.13	1.55 ± 0.09	0.47 ± 0.12	0.199	0.041
Betaine	1.63 ± 0.12	−0.07 ± 0.13	1.55 ± 0.14	0.31 ± 0.15	0.295	0.024
Carnitine	0.95 ± 0.11	0.22 ± 0.14	0.91 ± 0.09	−0.13 ± 0.11	−0.555	0.009
Citrate	11.49 ± 0.78	−1.15 ± 0.5	11.19 ± 1.16	0.53 ± 0.68	0.029	0.037
Formate	3.22 ± 0.28	0.01 ± 0.32	2.58 ± 0.19	1.31 ± 0.48	0.314	0.034
Glutamine	5.29 ± 0.29	−0.26 ± 0.24	4.61 ± 0.22	0.95 ± 0.22	0.220	0.0001
Glycine	10.51 ± 1.29	−0.83 ± 0.81	8.63 ± 0.75	1.18 ± 0.56	0.200	0.021
Histidine	4.33 ± 0.4	−0.55 ± 0.4	3.76 ± 0.34	1.25 ± 0.38	0.018	0.013
Lysine	1.83 ± 0.25	−0.27 ± 0.2	1.11 ± 0.1	0.3 ± 0.12	0.329	0.021
*N*-Phenylacetylglycine	3.05 ± 0.15	−0.16 ± 0.2	2.62 ± 0.13	0.53 ± 0.14	0.007	0.016
N6-Acetyllysine	1.04 ± 0.03	0.02 ± 0.04	0.99 ± 0.03	0.14 ± 0.04	0.001	0.028
Phenylacetate	1.04 ± 0.04	0.01 ± 0.05	1.07 ± 0.06	0.21 ± 0.06	0.002	0.021
Serine	5.19 ± 0.31	0.42 ± 0.35	4.05 ± 0.18	1.51 ± 0.31	0.248	0.021

GSH, reduced glutathione; GSSG, oxidized glutathione: GPx, glutathione peroxidase; Hb, hemoglobin; SOD, superoxide dismutase; MDA, malondialdehyde; IL-6, interleukin-6, TNF-α: tumor necrosis factor-alpha; KBR, Korean black raspberry. ^1^ Data are expressed as the means ± SEM; ^2^ The beta estimates (β; estimated slope) of each variable were determined using a linear mixed-effects model. The beta estimate describes the effect of the KBR group versus the placebo group on the linear change over the supplementation period; ^3^ Storey’s positive false discovery rate (pFDR) was calculated as *q*-values to account for multiple testing; ^4^ The absolute delta change was calculated by subtracting the measurement at baseline from that at the end of four weeks.

## Supplementary Materials

The following are available online at http://www.mdpi.com/2072-6643/9/3/233/s1, Figure S1: The Consolidated Standards of Reporting Trials flow diagram representing the phases of the randomized study for comparing the two species of raspberries, Table S1: Baseline characteristics of subjects participated in a preliminary study, Table S2: Daily dietary energy and nutrient intake at baseline and Week 4, Table S3: Comparison of anti-oxidant and anti-inflammatory effects of KBR and NAB in sedentary overweight/obese adults challenged with exercise, Table S4: Summary of urinary ^1^H NMR metabolites before and after placebo or KBR administration in sedentary overweight/obese adults challenged with exercise, Table S5: Summary of plasma ^1^H NMR metabolites before and after placebo or KBR administration in sedentary overweight/obese adults challenged with exercise, Table S6: Associations between the changes in traditional biomarkers and urinary metabolomic signatures at baseline.
