# Supplementary material for: Integration of Traditional and Metabolomics Biomarkers Identifies Prognostic Metabolites for Predicting Responsiveness to Nutritional Intervention against Oxidative Stress and Inflammation
Source: Nutrients. 2017 Mar 4;9(3):233. doi: 10.3390/nu9030233 (PMC5372896; doi:10.3390/nu9030233)
Supplement: Supplementary file 1 [file nutrients-09-00233-s001.pdf]

Supplementary Materials: Integration of Traditional and Metabolomics Biomarkers Identifies Prognostic Metabolites for Predicting Responsiveness to Nutritional Intervention against Oxidative Stress and Inflammation

You Jin Kim, Iksoo Huh, Ji Yeon Kim, Saejong Park, Sung Ha Ryu, Kyu-Bong Kim, Suhkmann Kim, Taesung Park, and Oran Kwon

3. Results

3.1. Preliminary Study to Compare Oxidative Stress and Inflammation in the KBR and NAB Groups Using Traditional Biomarkers

A total of 72 subjects were enrolled and 67 subjects were evaluable for response in a preliminary study (Supplemental Figure S1). All the participants were documented to fit the protocol and the groups were well matched for age and sex (Supplemental Table S1). From the three-day dietary records completed during the intervention, no significant group effect was detected across the baseline and four-week intervention among the dietary intake variables in terms of calories or micronutrients (Supplemental Table S2). The overall compliance was estimated at 96%. No serious or severe adverse events were.

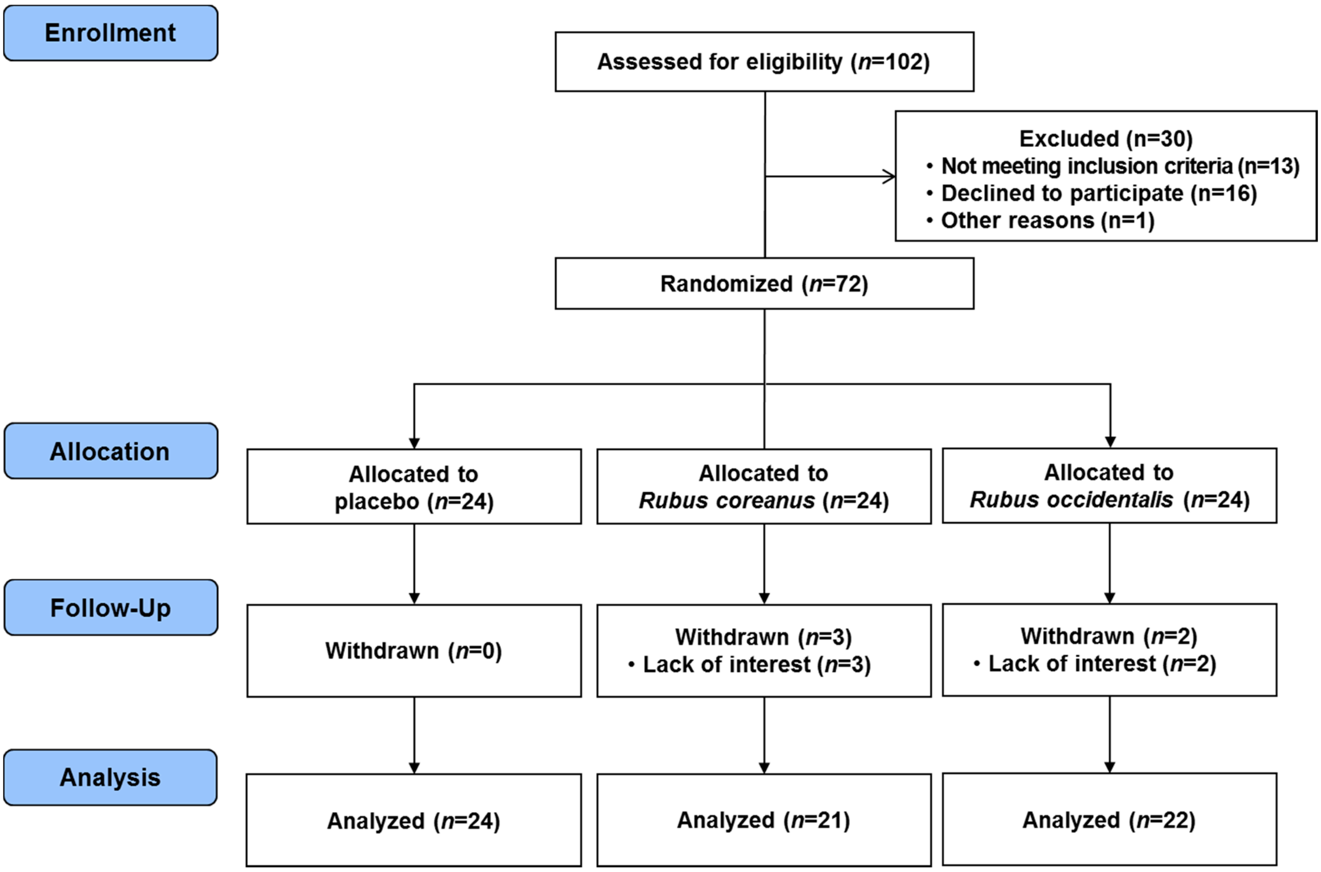

Figure S1. The Consolidated Standards of Reporting Trials flow diagram representing the phases of the randomized study for comparing the two species of raspberries.

Table S1. Baseline characteristics of subjects participated in a preliminary study [1].

Variables	Placebo (*n* = 24)	NAB (*n* = 22)	KBR (*n* = 21)
Age (y)	45 ± 8	42 ± 8	46 ± 7
Female/male (*n*)	13/11	13/9	10/11
BMI (kg/m^2)	25 ± 2	25 ± 2	25 ± 2
Body fat (%)	28 ± 8	27 ± 8	27 ± 8
WHR	0.87 ± 0.05	0.88 ± 0.06	0.87 ± 0.04
Blood pressure (mmHg)			
Systolic	130 ± 14	128 ± 8	129 ± 16
Diastolic	86 ± 12	86 ± 10	86 ± 23
FBG (mmol/L)	5.50 ± 0.61	5.44 ± 0.44	5.74 ± 1.00
Hb (g/L)	14 ± 1	15 ± 1	14 ± 2
Hct (%)	43 ± 3	44 ± 3	42 ± 4
VO_{2max} (mL/kg/min)	34 ± 6	35 ± 6	35 ± 7
HR_{max} (beat/min)	175 ± 13	178 ± 14	174 ± 12
BMR (kcal/day)	1574 ± 209	1608 ± 190	1550 ± 212
TEE (kcal/day)	2567 ± 403	2585 ± 393	2453 ± 467
Drinker/non-drinker (*n*)[2]	9/15	8/14	8/13

BMI: body mass index; BMR: basal metabolic rate; FBG: fasting blood glucose; Hb: hemoglobin; Hct: hematocrit; HRmax: maximum heart rate; KBR: Korean black raspberry; NAB: North American black raspberry; TEE: total energy expenditure; VO2max: maximum oxygen consumption; WHR: waist-to-hip ratio. 1 Data are expressed as the means ± SD for continuous variables or frequencies for categorical variables. 2 Drinkers were defined as participants who consumed one or more alcoholic drinks per month.

Table S2. Daily dietary energy and nutrient intake at baseline and Week 4 [1].

Variables	Placebo (*n* = 24)		NAB (*n* = 22)		KBR (*n* = 21)		*p*-value[2]		
	Baseline	Week 4	Baseline	Week 4	Baseline	Week 4	G	T	G*T
Energy (kcal)	1789 ± 438	1817 ± 372	1705 ± 656	1867 ± 653	1786 ± 407	1802 ± 436	0.783	0.149	0.402
Macronutrients (g)									
Carbohydrate	257 ± 66	258 ± 47	242 ± 65	258 ± 69	264 ± 56	252 ± 71	0.745	0.685	0.255
Protein	75 ± 35	72 ± 19	71 ± 34	74 ± 27	68 ± 17	73 ± 17	0.838	0.290	0.637
Fat	50 ± 16	51 ± 17	51 ± 31	58 ± 33	50 ± 16	52 ± 118	0.954	0.227	0.591
Minerals (mg)									
Calcium	529 ± 34	496 ± 44	504 ± 248	474 ± 174	558 ± 50	509 ± 49	0.593	0.201	0.580
Zinc	9.3 ± 1.0	9.0 ± 1.2	8 ± 3	8 ± 3	8.2 ± 0.5	8..5 ± 0.4	0.603	0.557	0.371
Iron	14 ± 1	12 ± 1	12 ± 5	12 ± 4	14 ± 1	13 ± 1	0.413	0.254	0.424
Vitamins									
Vitamin A (μg RE)	562 ± 45	640 ± 51	609 ± 288	587 ± 322	558 ± 40	621 ± 48	0.843	0.247	0.427
Retinol (μg)	111 ± 15	145 ± 24	94 ± 58	136 ± 120	120 ± 14	146 ± 29	0.540	0.081	0.723
β-Carotene (mg)	2.1 ± 0.2	2.5 ± 0.2	2621 ± 1494	2253 ± 1158	2.3 ± 0.2	2.5 ± 0.2	0.910	0.709	0.210
Vitamin E (mg)	12 ± 1	14 ± 1	12 ± 6	15 ± 10	13 ± 1	13 ± 1	0.824	0.045	0.324
Vitamin C (mg)	87 ± 11	72 ± 7	90 ± 66	63 ± 29	93 ± 13	89 ± 10	0.254	0.067	0.393
Folate (μg)	195 ± 15	211 ± 18	200 ± 84	194 ± 70	197 ± 12	214 ± 15	0.721	0.304	0.755

KBR: Korean black raspberry; and NAB: North American black raspberry. [1] All values are shown as the means ± SD. Intake levels were estimated from three-day food records using CAN-pro (Korean Nutrition Society, Seoul, Korea). The intake of the test material was not included in the analysis. [2] Repeated-measures ANOVA was used to compare the changes in nutritional intake over time (baseline and Week 4) between the three groups (placebo, NAB, and KBR). G, T, G*T represents main effects for group, time, and group X time interaction.

The MDA, oxidized LDL, TNF-α, and IL-6 were measured in plasma. However, endogenous antioxidants and enzymes including GSH, GSSG, GPx, SOD, and CAT were measured in erythrocytes, because they are abundant in erythrocytes, which are constantly subjected to oxidative stress. The data demonstrated that the overall effect was similar for both KBR and NAB, but KBR showed a more significant effect than NAB in terms of GSSG (q = 0.036), GSH:GSSG (q = 0.050), and MDA (q = 0.008) levels (Supplemental Table S3).

Table S3. Comparison of anti-oxidant and anti-inflammatory effects of KBR and NAB in sedentary overweight/obese adults challenged with exercise [1].

Variables	Placebo (n = 24)		NAB (n = 22)		KBR (n = 21)		Overall	PLA vs. NAB		PLA vs. KBR		NAB vs. KBR	
	Baseline	Delta change[2]	Baseline	Delta change	Baseline	Delta change	q-value[3]	β[4]	q-value	β	q-value	β	q-value
GSH (μM/g Hb)	34.0 ± 1.2	2.6 ± 0.7	35.2 ± 1.3	1.4 ± 0.6	33.7 ± 1.3	2.5 ± 1.0	0.333	-0.765	0.427	0.714	0.464	1.479	0.605
GSSG (μM/g Hb)	12.9 ± 0.6	1.8 ± 0.3	13.4 ± 0.6	0.7 ± 0.3	12.9 ± 0.7	0.3 ± 0.4	0.045	-1.023	0.049	-1.117	0.027	-0.095	0.036
GSH:GSSG ratio	3.0 ± 0.2	-0.3 ± 0.1	2.8 ± 0.2	-0.3 ± 0.2	2.8 ± 0.2	0.2 ± 0.2	0.116	0.025	0.250	0.045	0.039	0.021	0.050
GPx (nmol/min/g Hb)	60.8 ± 3.3	2.2 ± 1.2	60.9 ± 3.4	-0.7 ± 1.1	61.8 ± 3.5	4.1 ± 1.7	0.264	-0.017	0.332	0.012	0.480	0.029	0.465
SOD (U/g Hb)	21.8 ± 0.8	-0.4 ± 0.7	21.0 ± 0.8	-1.1 ± 1.0	21.8 ± 0.9	-0.3 ± 0.7	0.803	-0.011	0.611	0.003	0.897	0.014	0.803
Catalase (nmol/min/g Hb)	133.1 ± 5.4	-7.7 ± 3.9	129.8 ± 5.6	-12.2 ± 3.9	126.8 ± 5.8	-8.6 ± 5.3	0.284	-4.025	0.433	4.432	0.394	8.457	0.626
MDA (nM)	14.5 ± 1.5	0.0 ± 0.4	15.3 ± 1.6	-1.5 ± 0.4	16.4 ± 1.6	-2.5 ± 0.6	0.019	-0.037	0.067	-0.058	0.006	-0.020	0.008
Oxidized LDL (U/L)	46.0 ± 4.0	-0.8 ± 1.7	47.2 ± 4.2	-2.8 ± 1.3	46.8 ± 4.3	-5.3 ± 1.4	0.051	0.012	0.612	-0.044	0.060	-0.056	0.070
IL-6 (pg/mL)	196.1 ± 22.8	51.4 ± 31.8	177.0 ± 23.8	21.9 ± 17.1	182.7 ± 24.4	-52.1 ± 17.9	0.014	-0.056	0.407	-0.199	0.006	-0.143	0.185
TNF-α (pg/mL)	5.5 ± 0.5	0.6 ± 0.4	5.4 ± 0.5	0.7 ± 0.4	5.9 ± 0.5	0.1 ± 0.4	0.517	0.407	0.852	-0.547	0.279	-0.954	0.628

GSH, reduced glutathione; GSSG, oxidized glutathione: GPx, glutathione peroxidase; Hb, hemoglobin; SOD, superoxide dismutase; MDA, malondialdehyde; IL-6, interleukin-6; TNF-α, tumor necrosis factor-alpha. [1] Data are expressed as the means ± SEM. [2] The delta change was calculated by subtracting the measurement at baseline from that at the end of four weeks. [3] The q-values were calculated from p-values using Storey's false discovery rate approach. [4] The beta estimates (β; estimated slope) of each variable were determined using a linear mixed-effects model.

3.2. Selection of Traditional Biomarkers and Metabolites for Integration

The ^{1}H-NMR metabolomics data were obtained from the KBR group: 63 metabolites were identified in the urine samples (Supplemental Table S4) and the 31 metabolites were identified in the plasma samples (Supplemental Table S5). A LME model was used to assess differences in the KBR and the control group. As a result, four traditional biomarkers and sixteen urinary metabolites with FDR *q*-values less than 0.05 were selected to be included in further analysis (Table 1). Four traditional biomarkers were GSSG (q = 0.027) and GSH:GSSG ratio (q = 0.039) in erythrocytes and MDA (q = 0.006) and IL-6 (q = 0.006) in plasma; and sixteen metabolites were amino acids (alanine, asparagine, glutamine, glycine, histidine, lysine, serine, and carnitine), organic acids (citrate and formate), purine nucleotide (adenine), and other metabolites (N6-acetyllysine, betaine, 3-indoxylsulfate, N-phenylacetylglycine (PAG), and phenylacetate).

Table S4. Summary of urinary ^{1}H NMR metabolites before and after placebo or KBR administration in sedentary overweight/obese adults challenged with exercise [1].

Variables (M)	Placebo (n = 24)		KBR (n = 21)		β[3]	q-value[4]
	Baseline	Delta change[2]	Baseline	Delta change		
1-Methylnicotinamide	0.66 ± 0.06	0.09 ± 0.06	0.69 ± 0.05	0.02 ± 0.05	-0.001	0.299
2-Hydroxyisobutyrate	0.6 ± 0.03	-0.03 ± 0.02	0.54 ± 0.03	-0.05 ± 0.02	-0.033	0.421
3-Hydroxybutyrate	1.42 ± 0.1	-0.04 ± 0.11	1.17 ± 0.09	0.19 ± 0.11	0.137	0.164
3-Hydroxyisovalerate	0.65 ± 0.04	0.02 ± 0.03	0.55 ± 0.02	0.06 ± 0.03	0.028	0.590
3-Indoxylsulfate	2.31 ± 0.16	0.11 ± 0.21	2.16 ± 0.18	0.81 ± 0.2	0.399	0.009
3-Methyl-2-oxovalerate	0.91 ± 0.03	0.06 ± 0.04	0.81 ± 0.04	0.14 ± 0.05	0.001	0.206
4-Aminobutyrate	1.28 ± 0.06	0.08 ± 0.08	1.28 ± 0.07	0.12 ± 0.08	0.050	0.491
Acetate	0.62 ± 0.06	-0.01 ± 0.08	0.7 ± 0.11	-0.04 ± 0.09	-0.025	0.834
Acetylsalicylate	0.34 ± 0.02	0.13 ± 0.05	0.42 ± 0.04	0.01 ± 0.05	-0.148	0.287
Adenine	1.26 ± 0.11	0.09 ± 0.18	1.64 ± 0.21	-0.6 ± 0.2	-0.286	0.041
Alanine	2.79 ± 0.18	-0.14 ± 0.17	2.49 ± 0.16	0.35 ± 0.13	0.194	0.021
Arginine	2.83 ± 0.11	0.18 ± 0.14	2.45 ± 0.12	0.39 ± 0.16	0.002	0.258
Asparagine	1.62 ± 0.09	0.16 ± 0.13	1.55 ± 0.09	0.47 ± 0.12	0.199	0.041
Aspartate	2.59 ± 0.11	0.28 ± 0.14	2.32 ± 0.13	0.63 ± 0.14	0.003	0.084
Betaine	1.63 ± 0.12	-0.07 ± 0.13	1.55 ± 0.14	0.31 ± 0.15	0.295	0.024
Carnitine	0.95 ± 0.11	0.22 ± 0.14	0.91 ± 0.09	-0.13 ± 0.11	-0.555	0.009
Choline	0.56 ± 0.04	0.03 ± 0.04	0.5 ± 0.02	0.02 ± 0.03	0.000	0.904
Citrate	11.49 ± 0.78	-1.15 ± 0.5	11.19 ± 1.16	0.53 ± 0.68	0.029	0.037
Creatine	4.43 ± 0.5	-0.05 ± 0.52	4.07 ± 0.48	1 ± 0.88	0.108	0.405
Creatine phosphate	5.86 ± 0.36	1.07 ± 0.65	5.25 ± 0.45	1.7 ± 0.93	0.045	0.682
Dimethylamine	4.85 ± 0.37	1.39 ± 0.89	5.03 ± 0.27	1.29 ± 0.8	-0.005	0.956
Ethanol	1.2 ± 0.07	0.08 ± 0.09	1.04 ± 0.08	0.19 ± 0.08	0.001	0.355
Ethanolamine	5.83 ± 0.17	0.11 ± 0.19	5.79 ± 0.18	0.39 ± 0.26	0.003	0.344
Formate	3.22 ± 0.28	0.01 ± 0.32	2.58 ± 0.19	1.31 ± 0.48	0.314	0.034
Fucose	2.21 ± 0.12	-0.04 ± 0.13	2.04 ± 0.08	0.22 ± 0.09	0.100	0.163
Glucose	4.65 ± 0.18	0.31 ± 0.17	4.32 ± 0.31	0.44 ± 0.19	0.043	0.456
Glutamine	5.29 ± 0.29	-0.26 ± 0.24	4.61 ± 0.22	0.95 ± 0.22	0.220	<.0001
Glutamate	0.79 ± 0.04	0.09 ± 0.06	0.79 ± 0.04	0.06 ± 0.06	-0.043	0.631
Glutaric acid monomethyl ester	0.83 ± 0.04	0.07 ± 0.05	0.74 ± 0.04	0.13 ± 0.06	0.001	0.372

Table S4 (Continued)

Variables (M)	Placebo (*n* = 24)		KBR (*n* = 21)		β[3]	*q*-value[4]
	Baseline	Delta change[2]	Baseline	Delta change		
Glycerol	3.21 ± 0.1	0.48 ± 0.18	3.22 ± 0.18	0.42 ± 0.2	0.014	0.816
Glycine	10.51 ± 1.29	-0.83 ± 0.81	8.63 ± 0.75	1.18 ± 0.56	0.200	0.021
Guanidoacetate	6.22 ± 0.4	0.52 ± 0.29	5.83 ± 0.51	0.03 ± 0.56	-0.044	0.548
Hippurate	14.49 ± 1.57	-3.37 ± 1.92	16.55 ± 1.89	0.07 ± 2.16	0.034	0.157
Histidine	4.33 ± 0.4	-0.55 ± 0.4	3.76 ± 0.34	1.25 ± 0.38	0.018	0.013
Isobutyrate	0.49 ± 0.03	0.04 ± 0.03	0.49 ± 0.04	0.06 ± 0.02	0.071	0.284
Isoleucine	0.26 ± 0.01	0.01 ± 0.01	0.24 ± 0.01	0.04 ± 0.01	0.000	0.079
Lactate	6.63 ± 1.93	0 ± 3.57	4.13 ± 0.96	-0.75 ± 0.97	0.190	0.310
Leucine	0.45 ± 0.02	0.01 ± 0.02	0.42 ± 0.02	0.06 ± 0.02	0.000	0.084
Phenylacetate	1.04 ± 0.04	0.01 ± 0.05	1.07 ± 0.06	0.21 ± 0.06	0.002	0.021
Phenylalanine	1.03 ± 0.08	0.04 ± 0.11	1.13 ± 0.12	0.18 ± 0.12	0.001	0.480
Pyridoxine	0.34 ± 0.02	0.05 ± 0.05	0.39 ± 0.03	0 ± 0.03	-0.041	0.697
Pyruvate	0.76 ± 0.08	0.05 ± 0.09	0.67 ± 0.06	0.03 ± 0.05	-0.009	0.929
Serine	5.19 ± 0.31	0.42 ± 0.35	4.05 ± 0.18	1.51 ± 0.31	0.248	0.021
Succinate	0.45 ± 0.05	-0.02 ± 0.05	0.44 ± 0.03	-0.01 ± 0.04	0.084	0.460
Succinylacetone	0.81 ± 0.05	0.09 ± 0.06	0.74 ± 0.04	0.2 ± 0.06	0.001	0.171
Taurine	10.46 ± 0.66	2.12 ± 1.18	10.05 ± 0.6	0.73 ± 0.96	-0.101	0.395
Trigonelline	0.96 ± 0.11	0.13 ± 0.1	1.06 ± 0.13	0.1 ± 0.12	0.098	0.451
Trimethylamine	0.15 ± 0.01	0.02 ± 0.02	0.25 ± 0.04	0.13 ± 0.06	0.008	0.072
Trimethylamine N-oxide	6.4 ± 1.1	6.41 ± 3.78	7.2 ± 1.1	10.49 ± 5.56	0.290	0.245
Tyrosine	1.5 ± 0.08	0.11 ± 0.06	1.48 ± 0.06	0.23 ± 0.05	0.001	0.134
Uracil	1.25 ± 0.06	0.09 ± 0.07	1.35 ± 0.09	0.15 ± 0.11	0.001	0.602
Valine	0.61 ± 0.14	-0.12 ± 0.14	0.45 ± 0.02	0.05 ± 0.02	0.134	0.078
Xylose	1.16 ± 0.06	0.01 ± 0.09	1.43 ± 0.15	-0.07 ± 0.2	0.068	0.562
cis-Aconitate	2.67 ± 0.18	-0.09 ± 0.21	2.48 ± 0.12	0.31 ± 0.12	0.004	0.098
π-Methylhistidine	1.43 ± 0.22	0.73 ± 0.63	1.24 ± 0.17	0.26 ± 0.27	-0.103	0.610

[1] Data are expressed as the means ± SEM. [2] The delta change was calculated by subtracting the measurement at baseline from that at the end of four weeks. [3] The beta estimates (β; estimated slope) of each variable were determined using a linear mixed-effects model. [4] The *q*-values were calculated from *P*-values using Storey's false discovery rate approach.

Table S5. Summary of plasma ¹H NMR metabolites before and after placebo or KBR administration in sedentary overweight/obese adults challenged with exercise.[1]

Variables (M)	Placebo (n = 24)		KBR (n = 21)		β[3]	q-value[4]
	Baseline	Delta change[2]	Baseline	Delta change		
3-Hydroxybutyrate	2.31 ± 0.18	-0.29 ± 0.19	2.49 ± 0.22	-0.04 ± 0.24	0.069	0.909
Acetate	1.1 ± 0.08	-0.02 ± 0.09	1.2 ± 0.11	0.11 ± 0.12	0.084	0.909
Acetone	0.97 ± 0.07	-0.09 ± 0.07	1.1 ± 0.11	0.04 ± 0.1	0.071	0.909
Alanine	10.85 ± 0.8	-0.86 ± 0.76	13.3 ± 1.3	-1.66 ± 1.33	-0.074	0.909
Arginine	5.09 ± 0.34	0.13 ± 0.4	6.07 ± 0.54	-0.05 ± 0.69	-0.057	0.909
Betaine	2.42 ± 0.17	0.18 ± 0.21	2.97 ± 0.28	-0.2 ± 0.33	-0.120	0.909
Caffeine	0.45 ± 0.04	0.05 ± 0.04	0.52 ± 0.05	-0.01 ± 0.06	-0.160	0.909
Choline	1.57 ± 0.12	0.15 ± 0.12	1.72 ± 0.13	0.29 ± 0.22	-0.032	0.913
Citrate	5.54 ± 0.38	-0.49 ± 0.38	6.52 ± 0.59	-0.92 ± 0.64	-0.042	0.909
Creatine	1.16 ± 0.11	0.06 ± 0.09	1.47 ± 0.14	-0.21 ± 0.13	-0.157	0.909
Creatinine	0.86 ± 0.06	-0.03 ± 0.07	0.85 ± 0.05	0.06 ± 0.06	0.036	0.909
Ethylene glycol	2.33 ± 0.17	-0.24 ± 0.17	2.52 ± 0.22	-0.21 ± 0.27	0.002	0.987
Formate	0.9 ± 0.07	-0.04 ± 0.07	0.99 ± 0.08	0.02 ± 0.1	0.035	0.909
Glucose	118.56 ± 7.74	-6.82 ± 8.12	140.51 ± 11.64	-7.12 ± 12	-0.003	0.978
Glutamine	14.55 ± 1.07	-0.81 ± 0.98	17.18 ± 1.41	-1.62 ± 1.62	-0.071	0.909
Glycerol	7.57 ± 0.58	-0.67 ± 0.58	8.76 ± 0.78	-1.52 ± 0.84	-0.140	0.909
Glycine	5.97 ± 0.43	-0.41 ± 0.47	7.2 ± 0.64	-0.82 ± 0.66	-0.058	0.909
Histidine	2.36 ± 0.16	-0.16 ± 0.17	2.73 ± 0.22	-0.21 ± 0.24	-0.017	0.955
Isoleucine	2.27 ± 0.15	0.02 ± 0.17	2.58 ± 0.23	-0.12 ± 0.23	-0.062	0.909
Lactate	38.4 ± 5.15	-1.78 ± 5.07	43.6 ± 4.79	-7.56 ± 3.81	-0.195	0.909
Leucine	4.55 ± 0.3	-0.07 ± 0.31	5.1 ± 0.4	-0.16 ± 0.47	-0.030	0.909
Lysine	1.31 ± 0.1	0 ± 0.16	1.72 ± 0.2	-0.25 ± 0.25	-0.134	0.909
Methanol	2.18 ± 0.13	0.11 ± 0.17	2.37 ± 0.22	0.35 ± 0.25	0.131	0.909
N-Nitrosodimethylamine	3.04 ± 0.22	-0.03 ± 0.22	3.56 ± 0.32	-0.21 ± 0.38	-0.062	0.909
Phenylalanine	1.13 ± 0.08	-0.11 ± 0.08	1.35 ± 0.1	-0.15 ± 0.11	-0.044	0.909
Pyruvate	1 ± 0.11	0.11 ± 0.13	1.2 ± 0.13	0.08 ± 0.14	-0.109	0.909
Serine	5.14 ± 0.37	-0.1 ± 0.38	5.98 ± 0.54	-0.08 ± 0.66	-0.010	0.975
Succinate	0.38 ± 0.03	-0.01 ± 0.03	0.45 ± 0.04	-0.05 ± 0.04	-0.101	0.909
Threonine	5.98 ± 0.33	0.11 ± 0.43	7.59 ± 0.72	-0.09 ± 0.81	0.036	0.909
Tyrosine	1.69 ± 0.11	-0.03 ± 0.12	2.08 ± 0.16	-0.26 ± 0.17	-0.117	0.909
Valine	7.3 ± 0.48	0.1 ± 0.53	8.54 ± 0.7	-0.63 ± 0.71	-0.089	0.909

[1] Data are expressed as the means ± SEM. [2] The delta change was calculated by subtracting the measurement at baseline from that at the end of four weeks. [3] The beta estimates (β; estimated slope) of each variable were determined using a linear mixed-effects model. [4] The q-values were calculated from p-values using Storey's false discovery rate approach.

Supplementary Materials: The following are available online at http://www.mdpi.com/2072-6643/9/3/233/s1, Figure S1: The Consolidated Standards of Reporting Trials flow diagram representing the phases of the randomized study for comparing the two species of raspberries, Table S1: Baseline characteristics of subjects participated in a preliminary study, Table S2: Daily dietary energy and nutrient intake at baseline and Week 4, Table S3: Comparison of anti-oxidant and anti-inflammatory effects of KBR and NAB in sedentary overweight/obese adults challenged with exercise, Table S4: Summary of urinary ^{1}H NMR metabolites before and after placebo or KBR administration in sedentary overweight/obese adults challenged with exercise, Table S5: Summary of plasma ^{1}H NMR metabolites before and after placebo or KBR administration in sedentary overweight/obese adults challenged with exercise, Table S6: Associations between the changes in traditional biomarkers and urinary metabolomic signatures at baseline.

Table S6. Associations between the changes in traditional biochemical markers and urinary metabolomic signatures at baseline.[1]

Variables	GSSG			GSH:GSSG ratio			MDA			IL-6		
	β	t-value	*p*-value	*β*	t-value	*p*-value	*β*	*t*-value	*p*-value	*β*	t-value	*p*-value
3-Indoxylsulfate	-0.092	0.18	0.861	0.575	0.96	0.343	0	0	1.000	0.257	0.37	0.713
Adenine	0.397	-0.81	0.425	-0.876	-1.55	0.128	1.172	-2.47	0.018	0.314	0.48	0.633
Alanine	0.127	-0.26	0.799	-0.139	-0.25	0.805	0.652	-1.43	0.159	0.993	1.49	0.145
Asparagine	0.748	-1.52	0.136	-0.363	-0.65	0.520	0.652	-1.43	0.159	-0.464	-0.71	0.482
Betaine	0.396	-0.8	0.426	0.897	1.59	0.119	-0.277	0.62	0.540	-0.181	-0.28	0.784
Carnitine	0.193	-0.39	0.697	0.233	0.42	0.679	0.326	-0.72	0.473	0.792	1.21	0.234
Citrate	0.529	-1.06	0.295	0.763	1.36	0.181	0.149	-0.33	0.744	0.636	0.99	0.326
Formate	0.259	-0.52	0.605	0.617	1.09	0.280	0.005	-0.01	0.991	0.857	1.31	0.198
Glutamine	0.193	-0.39	0.697	0.233	0.42	0.679	0.530	-1.17	0.249	0.314	0.48	0.633
Glycine	0.394	-0.8	0.428	1.638	2.81	0.008	-0.277	0.62	0.540	-0.164	-0.25	0.803
Histidine	0.820	-1.66	0.105	0.246	0.44	0.664	0.124	-0.28	0.783	-0.387	-0.59	0.557
Lysine	0.391	-0.8	0.431	0.029	0.05	0.959	0.124	-0.28	0.783	1.295	1.95	0.057
N-Phenylacetylglycine	-0.157	0.32	0.754	1.820	3.08	0.004	-0.198	0.44	0.663	-0.599	-0.9	0.374
N6-Acetyllysine	-0.423	0.86	0.397	0.681	1.21	0.233	0.124	-0.28	0.783	-0.665	-1.01	0.319
Phenylacetate	0.053	-0.11	0.916	0.846	1.47	0.149	0.166	-0.37	0.716	-0.101	-0.15	0.879
Serine	0.131	-0.27	0.792	-0.351	-0.63	0.534	0.447	-0.99	0.328	0.240	0.37	0.716
Trimethylamine	0.867	-1.72	0.093	0.229	0.4	0.693	-0.127	0.27	0.785	-0.156	-0.23	0.817

[1] Analyses using a generalized linear mixed model with a logit link function (entry method) using the alterations of clinical biomarkers from blood as the dependent variable and baseline urinary ^{1}H NMR metabolomics data as the main predictor. The beta estimates (*β*) and the corresponding t-statistics (t-value) represent the magnitude of effect size (logit scale) and the test for significance for the respective independent variable. The exercise challenge was entered into the model as the confounding variable. Subjects with lower baseline metabolite values were the reference category.
